# Combined Ultrasound-Guided Thoracentesis, Percutaneous Pleural Biopsy, and Indwelling Pleural Catheter Insertion as the First Intervention in Patients With High Likelihood of Malignant Pleural Effusion

**DOI:** 10.1016/j.chpulm.2025.100201

**Published:** 2025-07-30

**Authors:** Michael V. Brown, Jelena Solujic, Sarah Yeo, Julia Kim, Phan Nguyen, Arash Badiei

**Affiliations:** a Department of Thoracic Medicine, Royal Adelaide Hospital, Adelaide, SA; b Faculty of Health and Medical Sciences, Adelaide Medical School, University of Adelaide, Adelaide, SA; c Respiratory and Sleep Medicine Unit, Austin Health, Melbourne, VIC; d School of Medicine, University of Melbourne, Melbourne, VIC, Australia

**Keywords:** closed pleural biopsy, indwelling pleural catheter (IPC), malignant pleural effusion (MPE), mesothelioma, ultrasound

## Abstract

**Background:**

Malignant pleural effusion (MPE) indicates advanced disease and imposes a significant symptomatic burden to patients. Current guidelines recommend stepwise investigation and management.

**Research Question:**

Is it feasible and safe to combine ultrasound (US)-guided pleural biopsy and indwelling pleural catheter (IPC) insertion as the initial diagnostic and therapeutic procedure for patients with high preprocedural probability of MPE?

**Study Design and Methods:**

We retrospectively analyzed patients who underwent pleural procedures between March 1, 2021, and September 30, 2022. Sixteen patients with symptomatic unilateral pleural effusion and clinical or radiologic features suggestive of malignancy underwent combined US-guided pleural biopsy and IPC insertion as their first management step. Feasibility was determined by the number of patients requiring repeat diagnostic and therapeutic procedures, and time to diagnosis. Safety was determined by complication rates.

**Results:**

Of 258 patients who received 384 pleural procedures, 16 patients (11 male; mean age ± SD, 77 ± 9.5 years) underwent the combined procedure. All patients had high preprocedural probability of MPE as evidenced by appropriate history, a unilateral pleural effusion (93.7%), and pleural nodularity or thickening on CT chest scan or US (87.5%). Mean time to diagnostic procedure was 9.3 days. Malignancy was confirmed in 100% of cases, with mesothelioma being the most common (50%). Pleural fluid cytology was diagnostic in 3 cases (18.8%), whereas 13 US-guided pleural biopsies (81.3%) were diagnostic. Nine patients (56.25%) had their IPC removed because of autopleurodesis or treatment response, with a mean removal time of 55.8 days. At 12 months, 5 patients (31.25%) had a documented complication, with pain and catheter blockage being the most common. One patient (6.25%) developed pleural infection. Over one-half (56.2%) received antineoplastic treatment with their IPC in situ. No patient required a repeat pleural procedure on follow-up.

**Interpretation:**

A combined approach of closed, percutaneous US-guided pleural biopsy and IPC insertion as initial pleural intervention was shown to be feasible in patients with high preprocedural probability for MPE with no unexpected safety signals.

**Take-Home Points**

**Study Question:** Is it feasible and safe to combine ultrasound (US)-guided pleural biopsy and indwelling pleural catheter (IPC) insertion as the initial diagnostic and therapeutic procedure for patients with high preprocedural probability of malignant pleural effusion (MPE)?

**Results:** Sixteen patients underwent the combined procedure. Pleural fluid cytology was diagnostic in only 18.8% of cases, whereas pleural biopsy was diagnostic in 81.3%. None of the patients required additional pleural procedures for the management of the MPE, and there was no signal to indicate increased complications.

**Interpretation:** In selected patients, a combined approach of closed percutaneous US-guided pleural biopsy and IPC insertion as initial pleural intervention was shown to be feasible with no unexpected safety signals.

Malignant pleural effusion (MPE) affects 5 million people worldwide annually.^1^ MPE affects 15% to 20% of patients with cancer and indicates advanced disease, often associated with dyspnea, weight loss, and functional decline.^2^^,^^3^ The presence of MPE carries a poor prognosis, with median survival ranging from 3 to 12 months.^3^

Initial investigation requires imaging. With widespread availability and progressively lower cost, CT scan is now commonly performed upfront, before pleural intervention. CT features supportive of MPE include nodular pleural thickening, mediastinal pleural thickening, parietal pleural thickening > 1 cm, and circumferential pleural thickening. These features have specificities of 94%, 94%, 88%, and 100%, and sensitivities of 51%, 36%, 56%, and 41%, respectively for malignancy.^4^, ^5^, ^6^ Thoracic ultrasound (TUS) can also help distinguish malignant from benign effusions and guide needle biopsy. Ultrasound (US) features that support malignancy include parietal pleural thickening > 1 cm, visceral pleural thickening, diaphragmatic thickening > 7 mm, and diaphragmatic nodules. Overall, US sensitivity for differentiating malignant from benign effusions is 79% with a specificity of 100%. The specificity compares favorably with CT scan (89%).^7^

The American Thoracic Society recommends pleural fluid aspiration for cytologic evaluation and symptom relief as the first step for suspected MPE.^8^ If cytology is nondiagnostic, stepwise investigation and management is recommended. The British Thoracic Society (BTS) has a similar algorithm also favoring initial aspiration. If nondiagnostic, thoracoscopy (medical or surgical) or radiologically guided pleural biopsy is recommended.^9^ The linearity of these existing pathways can lead to diagnostic delays in instances of nondiagnostic or insufficient samples, particularly relevant in the era of personalized cancer treatment.

US-guided percutaneous pleural biopsy is a bedside intervention which has higher diagnostic yield compared with fluid cytology and blind pleural biopsies, particularly when targeting pleural nodularity and thickening.^10^, ^11^, ^12^ Medical and surgical thoracoscopy are more invasive alternatives which have excellent diagnostic sensitivity,^13^^,^^14^ but have higher complication rates, have increased length of hospital stay, have increased health care cost, and are unsuitable for many patients with MPE.^13^^,^^14^ Management options for pleural fluid reaccumulation after intervention include repeat large volume thoracocentesis, chest drain insertion, indwelling pleural catheter (IPC), talc pleurodesis, or surgical management. These approaches have supporting evidence but performed separately result in multiple invasive procedures over weeks to months.

In select cases where the preprocedural probability of MPE is high, combination treatment with pleural fluid sampling, pleural biopsy, and IPC insertion has been associated with fewer subsequent procedures and lower health costs.^15^ The poor prognosis of MPE and substantial symptom burden demands a more streamlined diagnostic and therapeutic approach. This study investigates combining US-guided pleural biopsy and IPC insertion as the initial diagnostic and therapeutic intervention in patients with high preprocedural probability of MPE. Standard of care in our center includes all of these techniques in isolation or in combination at the discretion of the treating interventional pulmonologist. We aim to audit the safety and feasibility of the combined approach. Global literature in support of a combined approach is sparse, but we are aware that centers with pleural medicine expertise practice this.

## Study Design and Methods

All sequential patients who received pleural intervention presenting to the Royal Adelaide Hospital between March 1, 2021, and September 30, 2022, were retrospectively reviewed. Patients who received combined percutaneous US-guided pleural biopsy and IPC insertion as their first combined diagnostic and therapeutic procedure were analyzed.

**Figure 1:**
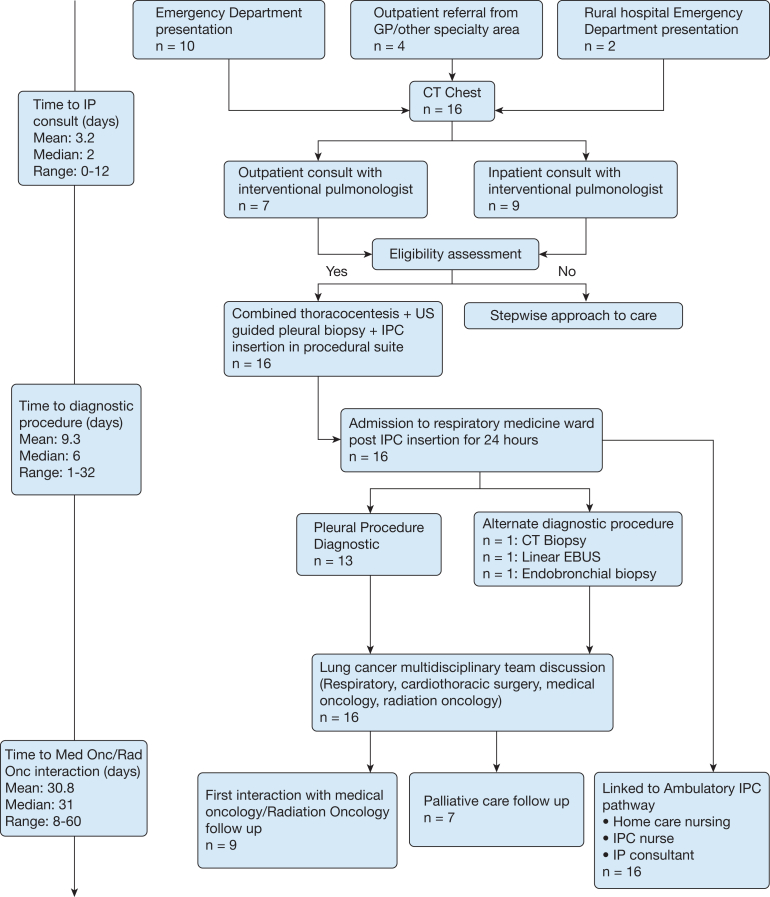
Combined thoracocentesis, US-guided biopsy, and IPC clinical pathway. EBUS = endobronchial ultrasound; GP = general practitioner; IP = interventional pulmonology; IPC = indwelling pleural catheter; Med Onc = Medical Oncology; Rad Onc = Radiation Oncology; US = ultrasound.

Symptomatic patients with unilateral pleural effusion with clinical and radiologic features suggestive of malignancy as deemed by an interventional pulmonologist were considered for combined intervention rather than a conventional stepwise approach. If there were clinical or radiologic uncertainty about underlying malignancy, or if there were contraindications to pleural biopsies (eg, bleeding diathesis) or IPC insertion, the patient was ineligible and received alternate guideline-based management (Fig 1).

### Clinical Features of Suspected Malignancy

Clinical features of suspected malignancy were the following: (1) active or past history of malignancy without prior evidence of pleural carcinomatosis or pleural effusion, (2) history of asbestos exposure with clinical and radiologic findings suggestive of pleural mesothelioma, (3) low likelihood of pleural infection based on clinical and biochemical features, and (4) absence of clinical and biochemical features suggesting congestive cardiac failure or liver cirrhosis complicated by portal hypertension and hepatic hydrothorax.

### Radiologic Features of Suspected Malignancy

Radiologic features of suspected malignancy are as follows: (1) radiologic (CT or 18F-fluorodeoxyglucose-PET scan) features of previously undiagnosed disseminated malignancy, (2) radiologic (CT scan) features of pleural malignancy (previously described),^16^ and (3) TUS features of pleural malignancy (previously described).^16^

Standard workup for an eligible patient included CT chest scan and TUS to help determine the likelihood of malignancy. Standard blood tests periprocedurally were also collected to ensure safety including complete blood examination, electrolytes, and coagulation. Once consented, the patient was positioned in the lateral decubitus position (pleural effusion side up). An initial TUS (Philips Sparq, C5-1 Curvilinear Probe with frequency range of 1-5 MHz) was performed to identify and characterize the pleural effusion, pleural thickening, or nodularity; identify the upper and lower effusion boundaries; and select an appropriate pleural biopsy site and IPC insertion site. Once patient positioning and TUS assessment were complete, procedural sedation using intravenous fentanyl and midazolam were administered. Supplemental oxygen was administered at a flow rate of 2 L/min according to local procedural sedation guidelines. Continuous vital sign monitoring (heart rate, blood pressure, and peripheral oxygen saturation) was maintained throughout the procedure.

Sterile skin preparation was then performed using a combination of alcohol and chlorhexidine (repeated 3 times) followed by sterile draping. Local anesthetic (lignocaine 1%) was infiltrated subcutaneously to the target areas. Further local anesthetic was then infiltrated to the parietal pleura and biopsy target under direct US guidance using a sterile probe cover. A 20-mL syringe with an 18-G needle was then used to take an initial pleural fluid sample for biochemistry analysis so that further pleural intervention would not alter the accuracy of the result. Then, under direct US guidance, biopsies (4-6) were performed using a Temno 6-cm 16-G or 18-G core needle (Medilogic, Sydney, NSW, Australia). The decision to proceed with pleural biopsy relied on the presence of pleural thickening or a nodular target. Biopsy needle gauge was based on proceduralist choice. All samples were sent in formalin for histologic processing.

A Rocket Medical 16-F IPC was then inserted and secured according to standard practice. Additional pleural fluid was collected for cytology (minimum 200 mL of pleural fluid) and cultures. Patients were admitted to hospital overnight for pleural fluid drainage, observation, and pain management. After hospital discharge, patients were linked with an IPC ambulatory care pathway. A home nursing service drained reaccumulated pleural fluid 2 to 3 times per week as dictated by patient symptoms. The primary contact for patients and home nursing services was an IPC nurse. The patient received ongoing 6- to 8-week medical follow-up through an interventional pulmonologist-lead pleural clinic.

Retrospective analysis of patient notes in the 12 months after management was analyzed to assess treatment course, complications, and whether repeat procedures were required. Adverse events were classed as detrimental events requiring specific medical intervention (eg, blood transfusion, ICU admission).

Feasibility was determined by the number of patients requiring repeat diagnostic and therapeutic procedures, and time to diagnosis. Safety was determined by complication rates. Secondary outcomes included diagnostic yield, defined as the number of patients in whom diagnostic samples were obtained divided by the total patients undergoing pleural intervention. Samples demonstrating histopathologic evidence of malignancy with adequate tissue for ancillary testing were diagnostic. Samples demonstrating atypia or other nonspecific findings were nondiagnostic and patients proceeded to alternate diagnostic procedures. This retrospective review was approved by the Central Adelaide Local Health Network Ethics and Governance Office (2024/HRE00090).

Statistical analysis was conducted using descriptive statistics. This included calculating frequencies and percentages for categorical variables (eg, sex distribution). For continuous variables, mean and SD were calculated to capture central tendency and dispersion, respectively.

## Results

**Figure 2:**
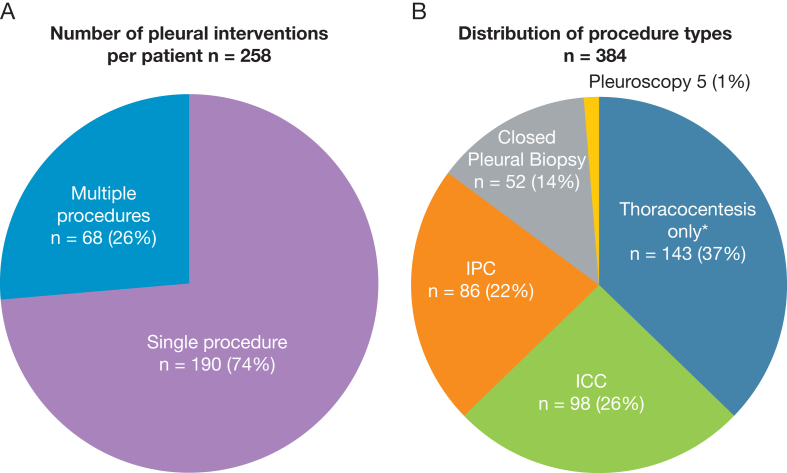
A, Number of pleural interventions per patient. B, Type of pleural interventions. Of the 258 patients, 16 patients had combined IPC and closed, percutaneous, ultrasound-guided closed pleural biopsy. ∗Thoracocentesis only: no other pleural intervention (eg, ICC or IPC insertion) was performed concurrently. ICC = intercostal catheter; IPC = indwelling pleural catheter).

Between March 1, 2021, and September 30, 2022, 384 pleural procedures were performed on 258 patients, including 143 thoracocenteses (37.24%), 98 intercostal catheters (25.52%), 86 IPCs (22.40%), 52 US-guided closed pleural biopsies (13.54%), and 5 pleuroscopies (1.30%). A total of 190 patients (73.64%) had a single procedure, whereas 68 patients (26.36%) required multiple pleural procedures (Fig 2). Sixteen patients (6.20%) underwent combined US-guided biopsy and IPC insertion, including 11 male individuals (68.7%) and 5 female individuals (31.3%). The average age ± SD was 77 ± 9.5 years. Seven patients (43.8%) had a prior malignancy history. Ten patients (62.5%) had a smoking history, and one-half (8 of 16; 50%) had prior asbestos exposure (Table 1). At initial presentation, 8 patients (50%) were Eastern Cooperative Oncology Group (ECOG) 0, 6 (37.5%) were ECOG 1, 1 (6.3%) was ECOG 2 and 1 (6.3%) was ECOG 3.

**Table 1:** Baseline Demographic Data and Baseline CT Scan Features (N = 16)

Demographic Data	Value
Sex	
Male	11 (68.75)
Female	5 (31.25)
Age	
Mean [SD]	77 [9.5]
ECOG at presentation	
0	8 (50.00)
1	6 (37.5)
2	1 (6.25)
3	1 (6.25)
Effusion location	
% right sided	10 (62.50)
Smoking history	
Previously or actively smoked	10 (62.50)
Prior asbestos exposure	
Yes	8 (50.00)
Prior malignancy history	
Yes	7 (43.75)
CT scan characteristics	
CT scan nodularity and/or thickening	14 (87.50)
Large pleural effusion	15 (93.75)
Moderate pleural effusion	1 (6.00)

Data are presented as mean [SD] or No. (%). ECOG = Eastern Cooperative Oncology Group.

Ten patients (62.5%) had their first health care interaction in the emergency department and were either admitted for inpatient care or discharged with short-term outpatient follow-up. Four patients (25%) were referred directly for outpatient management, and 2 patients (12.5%) were transferred from rural hospitals for tertiary center management. All patients had CT chest scan and concurrently or subsequently underwent outpatient (7 of 16) or inpatient (9 of 16) consultation to assess their malignancy risk and eligibility for a combined procedure. The mean time to interventional pulmonology consult was 3.2 days (median, 2 days; range, 0-12 days) (Fig 1). Radiologically, all patients (100%) had high preprocedural probability of malignancy. Fifteen patients (93.7%) had a large pleural effusion (more than one-half the base-apex length on Chest X-ray [CXR]), and 1 of 16 (6.3%) had a small to moderate pleural effusion (less than one-half the base-apex length on CXR). Fourteen patients (87.5%) had pleural nodularity and/or pleural thickening seen on CT scan. Ten patients (62.5%) had a right-sided pleural effusion (Table 1).

All 16 patients received a malignancy diagnosis. Eight patients (50%) were diagnosed with mesothelioma (biphasic: n = 4, sarcomatoid: n = 1, epithelioid: n = 1, not further classified: n = 2). Other diagnoses included 4 (25%) primary lung adenocarcinoma, 1 (6%) primary squamous cell carcinoma, 1 (6%) Waldenstrom Macroglobulinaemia, 1 (6%) metastatic gastrointestinal carcinoma and 1 (6%) melanoma. (Table 2). Pleural fluid cytology was diagnostic for malignancy in 3 patients (18.8%). Only one-third were satisfactory for ancillary testing, and two-thirds had their analysis deferred to the biopsy specimen. Pleural fluid cytology demonstrated atypia in 5 patients (31.3%) and was negative in 8 cases (50%). Conversely, 13 US-guided pleural biopsies (81.3%) were diagnostic for malignancy. Two biopsies (13.0%) demonstrated mild to moderate atypia suspicious of malignancy, and 1 case was nondiagnostic (Table 3). The nondiagnostic result occurred in a patient with multiple lung masses, a large effusion, and only mild pleural thickening without nodularity. In all instances of inconclusive pleural fluid cytology and pleural biopsy, an additional diagnostic procedure was performed and malignancy was confirmed. A linear endobronchial US biopsy of a mediastinal lymph node confirmed biphasic mesothelioma, a CT scan-guided lung biopsy confirmed a poorly differentiated primary lung adenocarcinoma, and a bronchoscopy with endobronchial biopsy confirmed squamous cell carcinoma. When ancillary testing was required for clinical management, the US-guided pleural biopsies were adequate in all cases (5 of 5; 100%). There were no complications from the additional 3 diagnostic procedures. The time to diagnostic procedure in all cases was a mean of 9.3 days, was a median of 6 days, and had a range of 1 to 32 days (Fig 1). The time to diagnostic procedure for patients with a nondiagnostic pleural biopsy/pleural fluid cytology was 18.7 days (range, 8-32 days).

**Table 2:** Method of Diagnostic Yield Per Histologic Diagnosis

Specific Diagnoses	Diagnostic on Cytology	Diagnostic on Pleural Biopsy	Diagnostic on Alternate Procedure
Biphasic mesothelioma	0/4 (0)	3/4 (75)	1/1 (100)
Sarcomatoid mesothelioma	0/1 (0)	1/1 (100)	NA
Epithelioid mesothelioma	0/1 (0)	1/1 (100)	NA
Mesothelioma (not further differentiated)	0/1 (0)	2/2 (100)	NA
Waldenstrom macroglobulinemia	0/1 (0)	1/1 (100)	NA
Metastatic gastrointestinal adenocarcinoma	1/1 (100)	1/1 (100)	NA
Melanoma	0/1 (0)	1/1 (100)	NA
Primary lung adenocarcinoma	2/4 (50)	3/4 (75)	1/1 (100)
Primary lung squamous cell carcinoma	0/1 (0)	0/1 (0)	1/1 (100)

Data are presented as No./total No. (%). NA = Not Applicable

**Table 3:** Diagnostic Yield Breakdown for Thoracocentesis and Pleural Biopsy

Diagnostic Data	No./Total No.	%
Thoracocentesis		
Exudative	15/16	93.75
Transudative	1/16	6.25
Cytology diagnostic	3/16	18.80
Cytology atypical	5/16	31.30
Cytology negative	8/16	50.00
Pleural biopsy		
Histology diagnostic	13/16	81.30
Histology atypical	2/16	13.00
Histology negative	1/16	6.00
Ancillary adequacy	5/5	100.00

On analysis of the 12-month period after the procedure, trapped lung was evident in 4 of 16 patients (25%). Nine patients (56.25%) had their IPC removed. In all cases, drainage output had diminished to a negligible volume (< 50 mL on 2 subsequent drainage attempts) for at least 2 weeks with clinical features suggestive of either autopleurodesis or treatment response. The mean time to drain removal ± SD was 55.8 ± 44.3 days. Five patients (31.25%) had their IPC in situ at time of death, and 2 patients (12.5%) were alive with functioning IPC at 12-month follow-up.

At 12-month post drain insertion, 11 patients (68.75%) did not have any complications. Five patients (31.25%) had at least 1 complication from their IPC. This included a blocked catheter in 1 patient (6.25%), which resolved with fibrinolysis with alteplase. One patient (6.25%) required readmission for pleural infection. This individual had been treated with symptomatic management only and no antineoplastic/immunosuppressive treatments. The patient declined antiinfective treatment, electing for palliative management and passed away 4 days later. Three patients (18.75%) had significant pain complicating pleural fluid drainage, and in all cases, the patients had alterations to their drainage regimens alleviating their symptoms. Nine patients (56.2%) had antineoplastic treatment with either immunotherapy or chemotherapy. The mean duration to a patient interacting with a medical oncologist after their original presentation was 30.8 days (median, 31 days; range, 8-60 days) (Fig 1). Seven patients (43.8%) had palliative intent with best supportive care (Table 4). No patient required repeated pleural procedures after the initial intervention.

**Table 4:** Follow-Up Outcomes at 12 Months

Follow-Up	Value
Complications	
Procedural complications	0 (0.00)
Blocked IPC catheter	1 (6.25)
Drainage related pain	3 (18.75)
IPC-related infection	1 (6.25)
No complications	11 (68.75)
Repeat pleural procedures required during follow-up	0 (0.00)
Cancer management postdiagnosis	
Immunotherapy	6 (37.50)
Chemotherapy/immunotherapy	2 (12.50)
Chemotherapy	1 (6.25)
Best supportive care	7 (43.75)
IPC outcomes	
IPC removed	9 (56.25)
Mean time [SD] to removal, d	55.8 [44.3]
In situ at time of death (still draining)	5 (31.25)
In situ alive (still draining)	2 (12.50)

Data are presented as mean [SD] or No. (%). IPC = indwelling pleural catheter.

## Discussion

This retrospective study evaluates the feasibility of combining US-guided pleural biopsies with IPC insertion. Patients with high preprocedural probability of malignancy underwent their diagnostic/therapeutic procedure within a mean of 9.3 days and median of 6 days. Although comparative literature is sparse, 1 multicenter retrospective cohort study reviewed MPE management pathway duration and reported a median time from first outpatient appointment (or emergency admission) to final diagnosis of 26 days (interquartile range [IQR], 14-48) days. In cases of negative cytology, patients had a median time to diagnosis of 30 days (IQR, 20-53) compared with 13 days (IQR, 9-17) in diagnostic cases.^17^ The time to diagnostic procedure was shorter in our study; however, delays were certainly present if the initial combined approach was nondiagnostic (mean, 18.7 days). The rapid diagnosis, particularly in mesothelioma cases, which can be historically complex to diagnoses,^18^ emphasizes this approach’s feasibility. Expediting workup and treatment is important because of the associations with survival, reduced anxiety, and better quality of life.

Based on our data, the combined approach appears safe because there were no procedural complications or need for repeat pleural procedures in the 12-month follow-up period. One-half of the cohort achieved autopleurodesis/treatment response and had their IPC removed in < 60 days. Overall complication rates were low, with all complications arising well after the initial procedure and in keeping with known IPC-related complications (eg, blockage, drainage pain, pleural infection).

Existing American Thoracic Society and BTS guidelines^8^ recommend stepwise management and investigation of suspected MPE. The first diagnostic step is thoracocentesis, which alleviates breathlessness but has limited diagnostic sensitivity between 46% and 67.2%.^19^, ^20^, ^21^, ^22^ Sensitivity varies by primary tumor type, being higher for lung adenocarcinoma (79%-88%) and lower for squamous cell carcinoma (14%-69%) and small cell carcinoma (44%-78%).^19^^,^^21^ Mesothelioma remains a challenging diagnosis with cytologic yields of 6% to 45.5%.^19^, ^20^, ^21^, ^22^, ^23^ Mesothelioma was well represented in this cohort, likely explaining our low thoracocentesis diagnostic yield. After an initial nondiagnostic result, second and third thoracocentesis have limited subsequent yields of 28.6% and 10.3%.^24^

Closed pleural biopsy, historically performed using an Abrams needle, has diagnostic sensitivity for malignancy of 27% to 60%, improving diagnostic yield over pleural cytology alone by 7% to 27%.^25^, ^26^, ^27^ A critical disadvantage of blind pleural biopsies is that malignant deposits are often close to the midline and diaphragm, increasing procedural risk. Image-guided (US or CT scan) pleural biopsy represents a safe alternative. US-guided needle biopsy was included in our workflow because it is a physician-led tool, not requiring a radiologist or general anesthetic. Meta-analysis of image-guided pleural biopsy has pooled diagnostic yields of 84% and 93% for US and CT scan-guided biopsy, respectively, with favorable complication rates of 3% and 7%.^10^, ^11^, ^12^^,^^28^

The patient selection for US-guided biopsy was based on existing literature of radiologic/tumor factors known to increase yield, including nodular morphology, greater pleural thickness, and needle pathway length (needle length through the pleural lesion). Diagnostic yield is as high as 96.4% in nodular lesions and 95% for diffuse lesions with > 4.5 mm of pleural thickness. Conversely, thickness < 4.5 mm is associated with a yield of only 55.6%.^10^ In cases of undiagnosed pleural exudates and in recurrent undiagnosed pleural effusions, US-guided pleural biopsy facilitates histopathologic diagnosis in 89.7% and 88.8% of cases, respectively.^29^^,^^30^

Local anesthetic thoracoscopy and video-assisted thoracoscopic surgery (VATS) can be a next step if the combined approach fails to manage or diagnose. Local anesthetic thoracoscopy has demonstrated benefit in patients with initial negative blind pleural biopsy.^31^ It has diagnostic sensitivity for MPE up to 92.6% and facilitates optional talc poudrage.^27^^,^^31^ The rate of major complications (empyema, hemorrhage, and pneumonia) is 2.3% and mortality is 0.40%. VATS also has high diagnostic sensitivity of 95% for malignancy with low complication rates.^27^^,^^32^ However, VATS has the disadvantage of requiring cardiothoracic expertise, a thoracic surgeon, general anesthetic, and single-lung ventilation and may therefore be unsuitable for certain patients. When comparing US-guided biopsy with medical thoracoscopy, the diagnostic accuracy for unexplained pleural effusion is 88.8% vs 96%, respectively. This slight improvement comes at the expense of complexity, health care costs, and morbidity, particularly with pain at the thoracoscopy port site.^33^ For these reasons, thoracoscopy is not first-line in our algorithm.

In cases specifically of suspected mesothelioma, BTS guidelines recommend pleural biopsy; however, a challenge for interventional pulmonologists is that radiographic features may be impossible to distinguish from nonmesothelioma pleural malignancy. Tsim et al^17^ evaluated proceeding directly to local anesthetic thoracoscopy in suspected mesothelioma cases and demonstrated that these patients were likely to have negative cytology and thus would benefit from upfront pleural biopsy in the first instance.

There is limited literature describing the suitability of pleural biopsy for molecular studies. As cancer therapies become increasingly personalized, improved sampling for molecular studies is essential. Tissue for molecular analysis is adequate in 95% of local anesthetic thoracoscopy, 86% of CT scan-guided biopsies, and 77% of US-guided biopsies.^34^ Conversely, pleural fluid alone is adequate for molecular studies in 61% of non-small cell lung cancer cases and 53% of overall MPE, mandating pleural biopsy in 65% of cases causing treatment delays.^17^ In some institutions, positive pleural cytology was only sufficient to guide management in 32% of cases, and most patients require further pleural procedures after initial thoracocentesis.^35^ These clear limitations of thoracocentesis highlight the value in up-front combined procedures.

The decision to use IPC for definitive pleural fluid management was also evidence-based in this cohort. IPCs improve symptoms in most patients (73%), providing equivalent relief to chest drains and talc pleurodesis, with added benefits of shorter hospital stay and reduced need for repeat procedures.^36^, ^37^, ^38^ Major society guidelines endorse IPC use, particularly for patients with nonexpandable (trapped) lung.^8^^,^^9^ IPC use does not increase morbidity, even in those on antineoplastic therapy. In the largest retrospective study to date, there was no difference in overall (6%-7%), deep pleural (3%-5%), or superficial (3%-4%) IPC-related infection rates between patients stratified by antineoplastic therapy or immune status.^39^ IPCs also facilitate talc instillation, increasing the probability of achieving pleurodesis.^40^

### Study Limitations

This is a retrospective review of an amalgamation of evidence-based procedures/management strategies that were combined into a single up-front procedure in highly selected patients. Patient enrollment and management was not protocolized, limiting generalizability. The selection of patients with high preprocedural probability of MPE may have led to a higher diagnostic yield, introducing selection bias. Small sample size represents another limitation reflecting the highly selective nature of patient suitability for this approach. Finally, although pleural-related readmission rates were included in our complication review, all-cause readmission was not captured within the limitations of this retrospective review.

Although each investigation and management step in this study has supporting evidence, the algorithm of up-front, combined pleural biopsy and IPC is not currently considered standard of care. Future prospective, multicenter studies comparing this combined approach with standard of care are needed to validate its effectiveness.

## Interpretation

Closed, percutaneous US-guided pleural biopsy and IPC insertion as the first combined pleural intervention was shown to be feasible in patients with a high preprocedural probability for MPE with no unexpected safety signals observed in this cohort. Time to diagnosis was short (mean, 9.3 days), and no patient required an additional pleural procedure in the follow-up period. This study suggests that a combined diagnostic and therapeutic approach using US-guided pleural biopsy and IPC insertion can be considered in select patients to facilitate timely diagnosis and early definitive management.

## Funding/Support

The authors have reported to *CHEST Pulmonary* that no funding was received for this study.

## Financial/Nonfinancial Disclosures

The authors have reported to *CHEST Pulmonary* the following: P. N. has received consulting fees from Pulmonx and Olympus Australia. None declared (M. V. B., J. S., S. Y., J. K., P. N., A. B.).
